# Relationship of Urinary Phthalate Metabolites with Serum Thyroid Hormones in Pregnant Women and Their Newborns: A Prospective Birth Cohort in Taiwan

**DOI:** 10.1371/journal.pone.0123884

**Published:** 2015-06-04

**Authors:** Fu-Chen Kuo, Sheng-Wen Su, Chia-Fang Wu, Meng-Chuan Huang, Jentaie Shiea, Bai-Hsiu Chen, Yi-Ling Chen, Ming-Tsang Wu

**Affiliations:** 1 Department of Public Health, Kaohsiung Medical University, Kaohsiung, Taiwan; 2 Department of Gynecology and Obstetrics, E-Da Hospital, Kaohsiung, Taiwan; 3 School of Medicine, College of Medicine, I-Shou University, Kaohsiung, Taiwan; 4 Department of Nutrition, Kaohsiung Medical University Hospital, Kaohsiung Medical University, Kaohsiung, Taiwan; 5 Department of Chemistry, National Sun Yat-Sen University, Kaohsiung, Taiwan; 6 Department of Laboratory Medicine, Kaohsiung Medical University Hospital, Kaohsiung Medical University, Kaohsiung, Taiwan; 7 Department of Nuclear Medicine, Kaohsiung Medical University Hospital, Kaohsiung Medical University, Kaohsiung, Taiwan; 8 Center of Environmental and Occupational Medicine, Kaohsiung Municipal Hsiao-Kang Hospital, Kaohsiung, Taiwan; 9 Department of Family Medicine, Kaohsiung Medical University Hospital, Kaohsiung Medical University, Kaohsiung, Taiwan; Institute for Health & the Environment, UNITED STATES

## Abstract

**Background:**

The purpose of this study was to examine the relationship of phthalates exposure with thyroid function in pregnant women and their newborns.

**Methods:**

One hundred and forty-eight Taiwanese maternal and infant pairs were recruited from E-Da hospital in southern Taiwan between 2009 and 2010 for analysis. One-spot urine samples and blood samples in the third trimester of pregnant women and their cord blood samples at delivery were collected. Nine phthalate metabolites in urine were determined by triple quadrupole liquid chromatography tandem mass spectrometry, whereas serum from pregnant women and their cord blood were used to measure thyroid profiles (thyroid-stimulating hormone [TSH], thyroxine, free thyroxine, and triiodothyronine) by radioimmunoassay.

**Results:**

Median levels of urinary mono-n-butyl phthalate, mono-ethyl phthalate, and mono-(2-ethyl-5-oxohexyl) phthalate (μg/g creatinine) were the three highest phthalate metabolites, which were 37.81, 34.51, and 21.73, respectively. Using Bonferroni correction at a significance of < 0.006, we found that urinary mono-benzyl phthalate (MBzP) levels were significantly and negatively associated with serum TSH in cord blood (β = -2.644, p = 0.003).

**Conclusions:**

Maternal urinary MBzP, of which the parental compound is butylbenzyl phthalate, may affect TSH activity in newborns. The alteration of thyroid homeostasis by certain phthalates in the early life, a critical period for neurodevelopment, is an urgent concern.

## Introduction

Phthalates are ubiquitously present in the environment; they are widely added to cosmetics as a vehicle for fragrance and to many other daily products, such as paints, children’s toys, and medical device to make them soft and flexible.[1] Because phthalates are easily released from these products, humans, particularly susceptible populations such as pregnant women and children, are potentially exposed to them via food ingestion, inhalation, or dermal absorption.

Phthalates are commonly thought to disrupt endocrine function and adversely affect sex and thyroid hormones, reproduction, and neurodevelopment.[2–5] The thyroid hormone is considered one of the important hormones to maintain normal physiological function in humans, especially for the fetus and newborn. Severe hypothyroidism will cause the retardation of growth and development and even the formation of cretinism.[3,6] Many *in-vitro* and *in-vivo* studies have shown that phthalates have functions similar to the thyroid hormone and the ability to bind thyroid receptors and, thus, affect thyroid homeostasis.[7–9] A few epidemiological studies, including ours, have investigated the relationship between exposure to different phthalates and the disruption of the thyroid profile in adults, adolescents, and children.[10] Although the findings of affected thyroid profile by certain phthalates are consistent, what and how particular phthalates chemically influenced what particular thyroid hormone is still inconclusive; in addition, only one study has reported the phthalates effect on thyroid function in pregnant women.[11–13] However, to our knowledge, no studies have examined whether phthalates exposure in pregnant women affects the thyroid function in their offspring after considering their own thyroid profile. Thus, we examined the relationship of maternal phthalate exposure by measuring their metabolites in urine with cord thyroid hormones after considering thyroid functions in pregnant women themselves.

## Materials and Methods

### Study subjects

This study followed the guidelines of STROBE.[14] We established one birth cohort in E-Da Hospital, a community teaching hospital located in southern Taiwan, to study the gene-environmental effect on pregnant women and their offspring since August 2009. Pregnant women attending E-Da Hospital for routine prenatal examinations were consecutively recruited. In order to establish a comprehensive biospecimen bank for the future study of gene-environment effect on offspring’s health, we started to recruit eligible pregnant women from their first trimester as the baseline. Potential study women were excluded if they had history of systematic diseases such as cancer, hypertension and diabetes, chronic use of corticosteroids or immunosuppressant drugs, and age was older than 45 years. Multiparous births were also excluded. Then, the eligible study women were interviewed by a standardized questionnaire and blood and one-spot urine specimens were collected and subsequently tracked during second and third trimesters, during delivery, and 2–3 weeks after delivery. In the four points of follow-up, besides routine prenatal examination, one additional diet short questionnaire and specimens of one-spot urine and blood were also collected. In addition, additional specimens of maternal stool, amniotic fluid, placenta, cord blood, and baby meconium were collected during delivery, whereas an additional specimen of breast milk was collected 2–3 weeks after delivery. The study protocol was reviewed and approved by the Institutional Review Board of E-Da hospital. Written informed consents were obtained from all study pregnant women for themselves and on behalf of their study children.

### Questionnaire

A standardized questionnaire in the first trimester was used to collect comprehensive information of demographic characteristics (age, height, weight, education, occupational history, medical care status, history of pregnancy, etc) and personal lifestyle habits (cigarette smoking, alcohol drinking, and areca nut chewing), diet and nutrient intake history, disease history, occupational history, history of drinking water, home and external environments such as house dampness, history of hair dyeing and cooking oil fume exposure, exercise activity, menstruation and history of pregnancy by a trained interviewer. The average length of interviews was approximately 30 minutes. The follow-up short questionnaire in the second and third trimesters, during delivery, and 2–3 weeks after delivery mainly focused on current diet and nutrient intake as well as the frequent use of different plastic products and melamine-containing tableware. It took ~10 minutes to finish the interview.

### Measurement of thyroid profile in serum

Samples of blood and cord blood were collected from pregnant women by phlebotomy in all three trimesters and umbilical cords of their newborns after delivery and immediately centrifuged at 4°C for 20 minutes. The supernatant of serum was aliquoted and stored at -80°C until analysis.

This study used the stored serum from the third trimester of pregnant women and serum from cord blood for the measurement of thyroid profile, including thyroid-stimulating hormone (TSH), thyroxine (T4), free thyroxine (FT4), and triiodothyronine (T3), by radioimmunoassay (RIA). The detailed analytical methods and their quality controls have been described previously.[15] In brief, RIA-gnost^®^ Cisbio Bioassays (Cisbio Bioassays International-CISBIO, Saclay, France) was used to measure thyroid profile in a clinical laboratory of the Department of Nuclear Medicine, Kaohsiung Medical University Hospital (KMUH), by one senior laboratory technician (YL Chen) who was blinded to the exposure status of study subject. The clinical laboratory was officially accredited by Taiwan Accreditation Foundation based on the accreditation criteria of ISO 15189:2007 (Certificate No. L1905-110705). The analytical sensitivity of radioimmunoassay was 0.03 μU/mL for TSH, 0.25 μg/dL for T4, 0.05 ng/dL for FT4, and 10.0 ng/dL for T3. All thyroid hormones in serum levels of mothers and cord blood could be detectable.

### Reagents

Mono-(2-ethylhexyl) phthalate (MEHP), mono-(2-ethyl-5-oxohexyl) phthalate (MEHHP), mono-(2-ethyl-5-hydroxylhexyl) phthalate (MEOHP), mono-n-butyl phthalate (MnBP), mono-iso-butyl phthalate (MiBP), monoethyl phthalate (MEP), mono-benzyl phthalate (MBzP), mono-methyl phthalate (MMP), mono-isononyl phthalate (MiNP) (all > 99.9%), and their ^13^C-labeled internal standards (> 99.9%) were purchased from Cambridge Isotope Laboratories, Inc. (Tewksbury, MA). Ammonium acetate (> 98%) was purchased from Sigma-Aldrich Laboratories, Inc. (St. Louis, MO). Ethyl acetate (99.9%) was purchased from J.T. Baker (Center Valley, PA). Methanol (MeOH), acetonitrile (ACN), formic acid (all > 98%), and water (HPLC-grade) were purchased from Merck (Taipei, Taiwan). β-glucuronidase was purchased from Roche Biomedical (Mannheim, Germany).

### Measurement of phthalate metabolites in urine

One-spot urine samples were also collected in a 15 mL polypropene (PP) tube in E-Da Hospital and immediately transferred and aliquoted into several 12 mL amber glass bottles. All the glassware was washed in MeOH, ACN and acetone, and then sealed with aluminum foil in order to prevent possible contamination of the urine samples from the environment. Urine samples were collected at the same time as serum samples, and all samples were stored at -20°C before analysis.

This study used the urine samples from the third trimester of pregnant women for the measurement of 9 phthalate metabolites, including MEHP, MEHHP, MEOHP, MnBP, MiBP, MEP, MBzP, MMP, and MiNP, by the method of liquid chromatography-tandem mass spectrometry (LC-MS/MS), slightly modified from Silva *et al*. (2004)[16] and described in details elsewhere.[17] The 9 phthalate metabolites included 6 primary metabolites (MEHP, MnBP, MiBP, MBzP, MEP, MMP, MiNP) of parent chemicals for di(2-ethylhexyl) phthalate (DEHP), di-n-butyl phthalate (DnBP), diisobutyl phthalate (DiBP), butylbenzyl phthalate (BBzP), diethyl phthalate (DEP), Dimethyl phthalate (DMP), and diisononyl phthalate (DiNP) and 2 secondary metabolites (MEHHP and MEOHP) of DEHP.

To prepare urine samples for analysis, 1 mL of each sample was thawed, sonicated, and poured into a glass culture tube, buffered with ammonium acetate (250μL, 1M, pH = 6.5) and then spiked with a mixture of isotope phthalate monoester standards (10 μL) and β-glucuronidase enzyme (3 μL, 200 U/mL). The samples were incubated in a 37°C water bath for 90 minutes. After hydrolysis, each sample was acidified by adding 2 mL phosphate buffer, vortex-mixed and centrifuged at 3,500 rpm for 10 minutes. The supernatant was loaded into a solid-phase extraction cartridge (NEXUS, Varian, Inc., Palo Alto, CA). Each 2 mL formic acid and water was added to remove hydrophilic compounds, and then 1 mL acetonitrile and ethyl acetate were added to elute metabolites. The combined elutes were concentrated under a stream of dry nitrogen at 55°C. Finally, the residues were reconstituted with water and subjected to LC-MS/MS for analysis.

### LC-MS/MS

LC-MS/MS analysis was carried out using an Agilent 1200 HPLC (Agilent Technologies, Palo Alto, CA, USA) coupled with an API 4000 Q triple-quadrupole mass spectrometry (API 4000 Q, Applied Biosystema/MDS SCEX, Concord, Canada) with an electrospray ionization (ESI) source in a negative ion mode. A 10 μL sample was injected into a ZORBAX SB-C18 column (250 × 4.6 mm, 5 μm, Agilent) at a flow rate of 1000 μL/min in the gradient mode from 80% mobile phase A (0.1% acetic acid in water) to 90% mobile phase B (0.1% acetic acid in acetonitrile). The TurboIon-Spray source was run at a temperature of 550°C with the following settings: curtain gas, 25 (arbitrary units); source gas 1, 50; source gas 2, 55; CAD gas pressure, medium; ion spray voltage, 5500. The precursor ion (*m/z*) and production ion (*m/z*) of 9 phthalate metabolites and 8 internal standard (IS) were as follows: 277 → 134 / 281 → 137 for MEHP; 293 → 121 / 297 → 124 for MEHHP; 291 → 121 / 295 → 124 for MEOHP; 221 → 77 / 225 → 79 for MnBP; 221 → 134 for MiBP; 193 → 77 / 197 → 79 for MEP; 255 → 183 / 259 → 107 for MBzP; 179 → 77 / 183 → 79 for MMP; 291 → 247 / 295 → 250 for MiNP (S1 Fig and S1 Table).

### Method validation

The calibration was performed by using standard solutions of phthalate metabolites in pooled urine samples. The corresponding rings labeled analogs were used as internal standard (IS). The calibration range of each metabolite was divided into two: 1–50 ng/mL for the low one and 50–1000 ng/mL for the high one. The correlation coefficients (R^2^) of these calibrations were 0.998–0.999 for MEHP, 0.996–0.998 for MEHHP, 0.998–1 for MEOHP, 0.994–0.998 for MnBP, 0.996–0.999 for MiBP, 0.991–0.996 for MEP, 0.993–0.999 for MBzP, 0.993–0.997 for MMP, and 0.998–1 for MiNP, which were all higher than 0.9950. Internal quality control was performed by analyzing both low (10 ng/mL) and high (100 ng/mL) levels spiked in urine in each batch. The accuracy for all calibration concentration curves and internal quality controls was within the range from 95.2 to 104.6% and with the precision expressed as a coefficient of variance (CV) ranging from 1.2 to 7.4% (n = 5). The intra- and inter-day relative standard deviation (RSD) ranged from 0.50–9.10% and 2.50–11.30% respectively. The averaged IS recovery in urine mixture was 80–115.0%, except for MEHP, MMP, and MiNP that showed IS recovery about 50%.

During the analyses of urine samples, each analytical run had 1–2 reagent blanks added, a low and a high concentration quality control sample, and 1–2 duplicated known samples. Calibration checks were also run every 20 samples to ensure instrumental stability throughout the entire analyses. Quantification of the calibration concentrations was within 15% of the theoretical value with a CV less than 15%, and therefore met performance criteria. The method of detection limit (MDL), determined by using a urine sample spiked with standard was 0.2 ng/mL for 5 phthalate metabolites (MEOHP, MEHHP, MBzP, MMP, and MiNP) and 1.0 ng/mL for others (MEHP, MnBP, MiBP, and MEP). The measurement below MDL was treated as half of MDL (S2 Table). Because MiNP was not detectable in all urine samples of our study subjects, we only presented the findings of the rest of the eight urinary phthalate metabolites.

### Creatinine analysis

For urinary creatinine, one spectrophotometry (U-2000, Hitachi, Tokyo, Japan) was used to measure the creatinine-picrate reaction at a wavelength of 520 nm. After corrected urinary creatinine, the phthalate metabolites were expressed as μg/g creatinine.

### Reconfirmation of phthalate-free urine containers

In order to confirm that these studied nine phthalate metabolites were not contaminated by the urine container (PP tube) or the external environment during the urine specimen collection in the clinic, we first prepared clean glass cups to collect one-spot urine samples from another volunteer and healthy females as controls. The eleven healthy females were from the preventive department for health check-ups, were willing to participate in this study, and their ages were similar to our study population.

Before use, the glass cups were washed and rinsed as mentioned above. None of the nine phthalate metabolites were detected in the pre-sampling glass cups. After one-spot urine samples were collected from the eleven healthy females in these clean glass cups, the cups were immediately covered with aluminum foil and transferred to the laboratory. Before analysis, each urine sample was halved with one half kept in the glass cup and the other half poured into a PP tube to simulate the same collection process used to collect urine samples from our study subjects. Urine samples contained by glass cups and PP tubes were simultaneously analyzed for the nine phthalate metabolites.^17^ We found that the median levels of studied urinary metabolites in the glass cups were not significantly different from those in PP tubes (All *p* values > 0.05, Wilcoxon signed rank test) (S2 Fig).

### Statistical analysis

Mean ± standard deviation (SD) or number (frequency) was tabulated to describe demographic characteristics, urinary metabolites, and serum thyroid profiles when appropriate.

Spearman correlation coefficients were first used to assess the correlation between eight urinary metabolites and serum thyroid profiles in pregnant women and their cord blood. When the *p*-value of any one urinary phthalate metabolite and any one thyroid hormone in cord blood reached < 0.05, its relationship was further examined in the multiple linear regressions to adjust for other significant phthalate metabolites also. Finally, in the full model, we included all the covariates, including age, gender, body mass index before the pregnancy (pre-BMI), weight gain during pregnancy, parity, education level, cigarette smoking, and alcohol drinking, as well as other phthalate metabolites, to test the robustness of the significant relationship between that particular phthalate metabolite and that particular thyroid index found in the significant crude analyses. The outcome variables of TSH, T4, FT4, and T3 in cord blood were log-transformed to normal distribution before the multivariate analyses.

Our prespecified research aim was to investigate whether the thyroid profile in cord blood was affected by phthalates exposure from the mother after considering serum thyroid profile in the mother and other covariates. Because the eight phthalate metabolites were studied, we used Bonferroni correction to express the two-sided significance at *p* < 0.006 (0.05/8). Data were analyzed using the SAS version 9.2 (SAS Institute Inc., Cary, NC).

## Results

### Demographic characteristics of participants

One hundred and forty-eight pairs of mothers and their newborns (92 and 56 male and female newborns) were consecutively recruited from August 2009 to December 2010 (Table 1). Mean age (± SD) of the participant mothers was 29.45 (± 4.93) years with a range of 16–42 years. The average pre-BMI and weight gain during pregnancy were 22.01 ± 3.55 kg/m^2^ and 13.01 ± 4.80 kg respectively. More than half of the participant mothers were experiencing their first pregnancy and had education levels ≥ college. Less than 5% of them had smoking and drinking habits during pregnancy (Table 1).

**Table 1:** Demographic characteristics of participant mothers and their newborns.

Variables N	Male 92	Female 56	Total 148
		Mean ± SD or N (%)	
***Mother***			
Age (years)	29.26 ± 5.04	29.51 ± 4.78	29.35 ± 4.93
Pre-BMI (kg/m^2^)	21.72 ± 3.23	22.48 ± 4.01	22.01 ± 3.55
Weight gain (kg)	12.73 ± 4.95	13.47 ± 4.55	13.01 ± 4.80
Parity			
1	48 (52.2)	28 (50.0)	76 (51.4)
2	39 (42.4)	24 (42.9)	63 (42.6)
3	5 (5.4)	2 (3.6)	7 (4.7)
4	0	2 (3.6)	2 (1.4)
Education level			
< college	40 (45.5)	21 (38.9)	61 (43.0)
≥ college	48 (54.5)	33 (61.1)	81 (57.0)
Cigarette smoking			
Yes	4 (4.4)	0	4 (2.7)
Drinking alcohol			
Yes	0	1 (1.8)	1 (0.7)
***Newborn***			
Gestation age (weeks)	38.44 ± 1.07	38.71 ± 1.18	38.54 ± 1.12
Birth height (cm)	50.16 ± 1.68	49.65 ± 1.89	49.97 ± 1.78
Birth weight (g)	3126.79 ± 340.45	3079.55 ± 336.17	3108.91 ± 338.47
Birth head circumference (cm)	33.85 ± 1.45	33.44 ± 1.38	33.70 ± 1.43
1-minute-Apgar score	7.95 ± 0.20	7.80 ± 0.86	7.89 ± 0.55
5-minute-Apgar score	8.96 ± 0.17	8.89 ± 0.45	8.93 ± 0.31

Abbreviation: SD = standard deviation; BMI = body mass index; Apgar = Appearance, pulse, grimace, activity, and respiration

The newborn ages were 38.54 ± 1.12 weeks of age with an average height and weight of 49.97 ± 1.78 cm and 3,108.91±338.47 g respectively (Table 1). The 1-minute- and 5-minute-APGAR (Appearance, Pulse, Grimace, Activity, and Respiration) scores were 7.89 ± 0.55 and 8.93 ± 0.31.

### Urinary phthalate metabolites

Median levels without creatinine correction (ng/mL) for 8 urinary phthalate metabolites were 7.71 for MEHP, 13.40 for MEOHP, 14.52 for MEHHP, 24.48 for MnBP, 13.21 for MiBP, 0.99 for MBzP, 22.49 for MEP, and 5.42 for MMP. After urinary creatinine correction, median levels for these 8 urinary phthalate metabolites (μg/g creatinine) were 11.92 for MEHP, 20.49for MEOHP, 21.73for MEHHP, 37.81for MnBP, 20.21for MiBP, 1.35for MBzP, 34.51for MEP, and 7.97for MMP (Table 2). Urinary MnBP, MEP, and MEHHP concentrations were the three highest recorded metabolites in this study (Table 2).

**Table 2:** Distribution of urinary phthalate metabolites and serum thyroid profiles in pregnant women and their cord blood serums.

Variables					Percentiles			
	N	Min	5th	25th	50th	75th	95th	Max
Urine (μg/g creatinine)								
MEHP	148	3.09	4.82	8.19	11.92	19.34	51.50	298.82
MEOHP	148	4.54	7.706	14.68	20.49	31.59	81.89	246.07
MEHHP	148	5.00	8.06	14.84	21.73	33.81	83.02	303.38
MnBP	148	8.85	13.52	26.47	37.81	58.96	131.38	663.03
MiBP	148	4.68	7.14	11.98	20.21	33.69	82.08	493.00
MBzP	148	0.23	0.44	0.86	1.35	2.68	8.10	57.05
MEP	148	2.85	6.48	17.92	34.51	88.60	328.00	9291.74
MMP^1^	148	ND	2.54	5.133	7.97	15.17	29.45	91.60
Pregnant women								
T3 (ng/dl)	148	106.00	123.00	149.00	168.00	192.00	234.00	350.00
T4 (μg/dl)	148	4.30	7.90	9.80	10.90	12.00	13.70	17.40
FT4 (ng/dl)	148	0.81	0.85	1.03	1.10	1.16	1.29	1.66
TSH (μIU/ml)	148	0.001	0.45	0.90	1.33	1.70	3.33	4.73
Cord blood								
T3 (ng/dl)	148	35.00	41.00	50.00	56.00	62.00	75.00	125.00
T4 (μg/dl)	148	6.60	8.00	9.40	10.55	11.45	13.10	15.30
FT4 (ng/dl)	148	0.86	0.91	0.99	1.08	1.16	1.32	1.65
TSH (μIU/ml)	148	1.51	2.98	4.20	5.54	8.34	17.65	25.42

Abbreviations: Max = Maximum; Min = Minimum; MEHP = Mono-(2-ethylhexyl)phthalate; MEOHP = Mono-(2-ethyl-5-hydroxylhexyl) phthalate; MEHHP = Mono-(2-ethyl-5-oxohexyl) phthalate; MiBP = Mono-iso-butyl phthalate; MnBP = Mono-n-butyl phthalate; MBzP = Mono-benzyl phthalate; MEP = Mono-ethyl phthalate; MMP = Mono-methyl phthalate; MiNP = Mono-isononyl phthalate; TSH: Thyroid-stimulating hormone; T4: Thyroxine; FT4: Free thyroxine; T3: triiodothyronine.

^1^ND in 3 urine samples were not detected.

### Serum thyroid profiles

All serum thyroid profiles in pregnant women and cord blood were detectable, and are summarized in Table 2. Median levels of T3, T4, Free T4 and TSH in maternal serum were 168.00 ng/dL, 10.90 μg/dL, 1.10 ng/dL, and 1.33 μIU/mL, whereas median levels of T3, T4, Free T4 and TSH in cord blood serum were 56.00 ng/dL, 10.55 μg/dL, 1.08 ng/dL and 5.54 μIU/mL.

### Relationship between urinary phthalate metabolites and thyroid hormones

**Fig 1:**
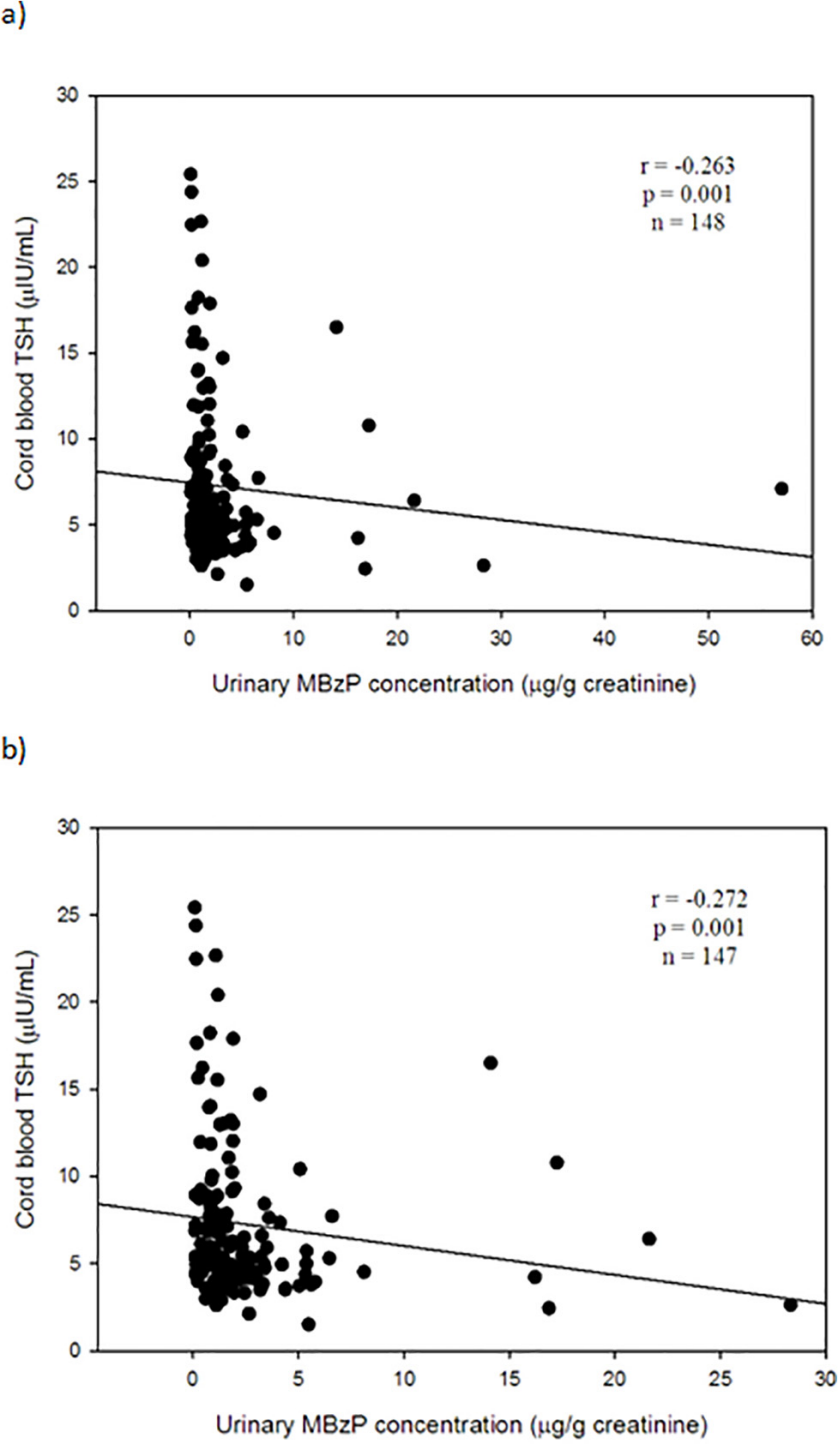
Relationship between urinary MBzP levels and TSH levels in cord blood serum. a) In total (n = 148); b) One outlier exclusion (n = 147). Abbreviation: MBzP = mono-benzyl phthalate; TSH = thyroid-stimulating hormone.

Table 3 shows any correlations between urinary phthalate metabolites in pregnant mothers and thyroid profiles in maternal and cord blood serum. We found that urinary MBzP in pregnant mothers was the only one to be significantly associated with TSH levels in cord blood serum at *p*-value level < 0.05 (Tables 3 and 4). Urinary MBzP was significantly and negatively associated with TSH levels in cord blood serum (Spearman correlation coefficiency, r = -0.263, n = 148, p = 0.001) (Fig 1A). This significant finding was not changed after excluding one outlier (Fig 1B). We did not find any significant association between any of all eight urinary phthalate metabolites and T4, FT4, or T3 in cord blood serum at level of < 0.05. After adjusting for other covariates in the full model, urinary MBzP levels were still significantly and negatively associated with TSH levels in cord blood serum (β = -2.604, p = 0.002) with Bonferroni correction at two-sided *p* < 0.006 (Table 4). No association between any of all other seven urinary phthalate metabolites and T4, FT4, or T3 in cord blood serum at significant level of < 0.006 was noted (data not shown) (Fig 1, Table 3, Table 4).

**Table 3:** Spearman correlation between urinary phthalate metabolites and serum thyroid profiles in pregnant women and cord blood.

									Pregnant women				Cord blood			
	MEHP	MEOHP	MEHHP	MnBP	MiBP	MBzP	MEP	MMP	T3	T4	FT4	TSH	T3	T4	FT4	TSH
**Urinary phthalate metabolites**																
MEHP	1.0															
MEOHP	**0.715** *	1.0														
MEHHP	**0.775** *	**0.904** *	1.0													
MnBP	**0.264** *	**0.257** *	**0.235** *	1.0												
MiBP	**0.316** *	**0.280** *	**0.266** *	**0.688** *	1.0											
MBzP	**0.247** *	**0.264** *	**0.282** *	**0.310** *	**0.276** *	1.0										
MEP	0.045	0.088	0.012	**0.266** *	**0.271** *	0.117	1.0									
MMP	**0.216** *	**0.195** *	0.138	0.090	**0.204** *	-0.026	0.091	1.0								
**Pregnant women**																
T3	-0.084	0.053	-0.101	-0.012	-0.073	0.068	0.025	-0.055	1.0							
T4	0.059	0.131	0.062	0.098	0.004	**0.209** *	-0.126	-0.149	**0.521** *	1.0						
FT4	0.127	**0.225** *	0.208	**0.167** *	-0.046	**0.241** *	-0.037	-0.137	**0.178** *	**0.611** *	1.0					
TSH	-0.122	-0.101	-0.155	-0.103	-0.055	-0.130	0.011	0.065	-0.106	-0.091	-0.129	1.0				
**Cord blood**																
T3	-0.074	-0.079	-0.058	0.018	0.084	-0.002	-0.025	-0.070	0.079	-0.027	-0.134	-0.100	1.0			
T4	0.000	0.065	0.039	0.117	0.128	0.131	-0.033	0.038	**0.301** *	**0.237** *	0.014	-0.091	**0.644** *	1.0		
FT4	-0.047	-0.070	-0.051	-0.020	0.076	0.015	0.016	0.139	0.034	-0.126	**-0.171** *	-0.050	**0.408** *	**0.552** *	1.0	
TSH	-0.268	**-0.221** *	**-0.263** *	-0.080	0.042	**-0.263** *	-0.030	-0.023	0.009	-0.036	-0.118	0.138	**0.270** *	0.125	0.089	1.0

Abbreviation described in Table 2.

*p-value <0.05.

**Table 4:** Relationship of thyroid stimulating hormone (TSH) in cord blood with urinary phthalate metabolites in pregnant women in crude and full models.

Variables	Log-transformed TSH in cord blood			
	Model 1		Model 2*	
	Crude	*p*-value	Adjusting	*p*-value
**Pregnant women**				
Age	-0.099	0.198	-0.109	0.206
Gender (female *vs*. male)	0.591	0.451	0.088	0.914
Pre-BMI	-0.08	0.460	0.037	0.758
Weight gain	0.096	0.230	0.062	0.483
Gestational age	-0.031	0.362	0.179	0.656
Parity	0.146	0.803	-0.078	0.895
Educational level (≥ college *vs*. < college)	-0.469	0.178	-0.529	0.151
Cigarette smoking (yes *vs*. no)	-2.362	0.305	-1.773	0.467
Alcohol drinking (yes *vs*. no)	-3.126	0.493	-0.469	0.322
TSH	0.366	0.428	-0.047	0.923
**Urinary phthalate metabolites**				
MEHP	-2.284	0.055	-1.342	0.541
MEHHP	-2.266	0.071	-2.375	0.575
MEOHP	-2.315	0.060	2.676	0.499
MnBP	-2.079	0.090	0.521	0.761
MiBP	-2.016	0.070	-1.078	0.482
**MBzP**	**-2.644**	**< 0.0001**	**-2.604**	**0.002**
MEP	-1.342	0.060	-1.242	0.121
MMP	0.848	0.473	1.852	0.147

*All listed variables in the model.

## Discussion

The present study found that the higher the urinary MBzP levels in pregnant mothers, the lower the TSH levels in cord blood serums. To our knowledge, this is the first report to examine the association of maternal phthalate exposure and thyroid hormones in cord serum samples after considering maternal thyroid function.

A series of animal studies have suggested that phthalates can disrupt thyroid function through the pathways of: 1) Affecting the T3 binding to transport proteins; 2) Interacting with the uptake of active T3 in plasma membrane; 3) Acting as an antagonist at the thyroid hormone receptors (TR), e.g., competing to bind with transthyretin (TTR) and further inhibiting the expression of thyroid hormone receptor beta (TR-β) gene; and 4) Affecting the transcriptional activity of sodium/iodide symporter (NIS) or TR, etc.. [7,18–21] By contrast, studies of pregnant animals concerning thyroid toxicity of phthalates are scarce, although the reviewed articles have considered their adverse effect on thyroid in pregnant animals and their offspring.[2,3]

Once in the human body, phthalates are rapidly metabolized by hydrolysis and subsequent oxidation reactions. Phthalates metabolites are almost completely excreted via urine with excretion completed within a day or two.[22] For short-chain phthalates such as DnBP, DiBP, BBzP, DEP, or DMP, simple monoesters (primary metabolites) are the major urinary metabolites, with urinary excretion of these compounds representing up to 70% of their oral doses.[23] For long chain phthalates such as DEHP and DiNP, simple monoesters are further metabolized to produce a variety of oxidative metabolites and only 2–7% of the total absorbed dose is excreted as a simple monoester.[24–26] As a consequence for biomonitoring studies in humans, the concentrations of monoester metabolites in urine accounts well for exposure to short chain phthalates, whereas secondary oxidized metabolites measurements are more suitable to assess the exposure to long chain phthalates, which was the reason that we measured the primary metabolites for DnBP, DiBP, BBzP, DEP, and DMP and additional secondary metabolites for DEHP as the proxies of phthalates exposure in pregnant women and their newborns in this study.

In this study, we found that levels of urinary MEHHP, MnBP and MEP were the highest ones of the nine metabolites measured, which indicated that the participants were exposed predominantly to the parental chemicals of DEHP, DBP and DEP. In contrast, MiNP were not detectable in all urine samples, suggesting it may not be an appropriate proxy for DiNP exposure. The secondary metabolites of DiNP such as monohydroxyisononyl phthalate (MHiNP) and monooxoisononyl phthalate (MOiNP) can be used as additional proxies for DiNP exposure.[11] Alternatively, external DiNP exposure in this study population may be neglected. Compared to the measures of previous studies from Taiwan, European countries, and the USA, the studied phthalate metabolites were not higher (S3 Table).[13,27–37] By contrast, the phthalates metabolites were not as low as those in a Japan study.[38]

A few human studies, including ours, have examined the relationship between phthalates exposure, particularly DEHP, and serum thyroid profiles in adults, adolescents, or children (S4 Table).[10,11,15,39] Meeker and his coworkers firstly studied 408 men and collected their blood and one-spot urine samples when they visited one Fertility Center in Massachusetts, USA, for infertility evaluation.[10] They measured six phthalate metabolites, including MEP, MBP, MBzP, MEHP, MEHHP, and MEOHP in urine and TSH, FT4, and T3 in serum. They found a significantly inverse association between MEHP and serum FT4 levels, but not TSH and T3, after adjusting for other covariates. In contrast, MEHHP was significantly and positively associated with FT4, but not TSH and T3, in a subgroup of 208 study men. Other phthalate metabolites were not significantly associated with thyroid profiles. Boas and colleagues measured 12 phthalate metabolites from one-spot urine samples and serum thyroid profiles including TSH, T3, T4, FT3, and FT4, in 845 Danish children aged 4–9 years.[11] The 12 phthalate metabolites included MEP, MnBP, MBzP, MEHP, MEHHP, MEOHP, mono (2-ethyl-5-carboxypentyl) phthalate (MECPP), mono-*n*-octyl phthalate (MOP), MiNP, MHiNP, MOiNP, and monocarboxyisooctyl phthalate (MCiOP). They found that only MEP was negatively and significantly associated with T3 in girls. Recently, Meeker & Ferguson (2011) studied 1,346 adults whose age ≥ 20 years and 329 adolescents aged 12–19 years from the 2007–2008 National Health and Nutrition Examination Survey (NHANES). One-spot urine was used for measuring seven metabolites of DEHP (MEHP, MEHHP, MEOHP, and MECPP), DBP (MnBP, MiBP, and mono (3-carboxypropyl) phthalate (MCPP)), whereas TSH, T4, FT4, T3, and thyroglobulin were measured in serum. They found that only MEHHP of DEHP metabolites displayed monotonic dose-dependent decreases in T4, but not other thyroid profiles. In addition, all four DEHP metabolites were not significantly associated with TSH levels. In adolescents, they observed a significant and positive association between DEHP secondary metabolites and T3 and TSH. Overall, no significant associations were noted between DBP metabolites and thyroid profiles. Although these studies suggest DEHP metabolites may disrupt the thyroid signaling in adults and children, the alteration of which particular thyroid hormone by which phthalate metabolite was inconsistent. In addition, all the above significant findings at levels < 0.05 were necessarily interpreted cautiously due to the bias of multiple comparisons for type-I error.

In the earliest study, Rais-Bahrami *et al*. (2004) examined thyroid profiles in 19 adolescents (13 males and 6 females) aged 14–16 years who were exposed to a relatively high DEHP concentration (~42–140 mg DEHP/kg body weight/day for 3–10 days) through intravenous blood exchange transfusions by using extracorporeal membrane oxygenation (ECMO) during the neonate stage (S4 Table). They found that no significant adverse effects of DEHP on the physical growth and pubertal maturity when they become adolescents. Besides normal function of liver, renal, and male and female gonadal systems, thyroid function, including TSH, T4, and FT4 levels in serum was within the normal range of age- and sex-distribution. The discrepancy of this study compared to the subsequent studies[10,11,15,39] is speculative due to the lack of significant conversion of DEHP to MEHP under acute and short-term DEHP exposure in an intravenous form.[40]

On April-May of 2011, a major incident of phthalate-contaminated foodstuffs occurred in Taiwan.[41] Phthalates, mainly DEHP, were deliberately added to foodstuffs, particularly health food or supplements in tablet or powder form for children, as a substitute of emulsifier.[15] We collected 60 children who visited our special children clinic for phthalate exposure in southern Taiwan between May 31 and June 17, 2011. By interviewing the main caregivers of affected children to construct the exposure matrix of DEHP from contaminated foodstuffs, we found that the higher the DEHP exposure, the more significant the lower serum TSH levels.[15] Our study showed that serum TSH activity could be altered when children were exposed to high concentrations of phthalate-tainted foodstuffs, although the sample size was small.

The data regarding phthalates effect on thyroid function in pregnant women are scarce.[11–13] In 2007, Huang and coworkers analyzed phthalate exposure and thyroid hormones in 76 Taiwanese pregnant women at second trimester. They measured five urinary phthalate monoesters, including MnBP, MBzP, MEHP, MEP, and MMP and thyroid hormones, including TSH, T3, T4, and FT4. They found that urinary MnBP levels were negatively and significantly associated with T4 and FT4 (*p* = 0.003 and < 0.001 respectively). From this study, we found that the studied pregnant women were those where the need to undergo amniocentesis by gynecologists had been suggested, including age older than 35 years or abnormal serum levels of α-fetoprotein or β-human chorionic gonadotropin. Thus, generalizability to normally pregnant women is a concern. In addition, information about thyroid function in cord blood is lacking.

There was one outlier with high urinary MBzP level in this study. We checked the questionnaire of that outlier and found that that particular woman consumed high-temperature food covered by polyvinyl chloride (PVC) films during pregnancy daily. One previous study has reported that PVC films were one of the main sources of phthalates exposure when in contact with high-temperature foods in the Taiwanese population.[42]

The present study has some limitations. One was the collection of only one-spot urine samples to measure phthalate metabolite levels. The random variation of exposure variable may be underestimated in our findings. Another limitation is that the reference ranges of thyroid hormone profiles were not established for both third-trimester pregnant women and their cord bloods in Taiwan; therefore, we were unable to explore whether all the measures in this study were or were not within the normal ranges. The findings of this study are from one Taiwanese population; thus, generalizability to other populations is cautioned. Furthermore, the appropriateness of using urinary creatinine to adjust for phthalate metabolite levels in pregnant women is unknown, because creatinine can be influenced by muscle mass, racial differences, and dietary intake of meat.[13]

Our study also found that usage of plastic or glass vials to collect urine would not affect the level of phthalate metabolites measured in the urine. In the previous study, it was recommended glass vials be used to collect and store urine samples.[13] Because the use of plastic containers can reduce cost and make transportation and storage processes more convenient in busy clinical settings, the current result, plus our previous finding, suggests PP-made containers can be used for urine collection without affecting the accuracy of urine metabolite measurement.[15]

In summary, the present study found the higher the urinary MBzP level in pregnant mothers, the lower the TSH level in cord blood serum. The parental compound of MBzP is BBzP, which is commonly used as a plasticizer for vinyl foams and is often used in the manufacture of floor tiles. Although the study found that the influence of TSH levels in the cord blood is still within the normal physiological range, the disruption of normal thyroid homeostasis in the early life has been reported to be crucial for the development of central nervous system. Thus, we should realize that this critical phase may be vulnerable to even subtle changes of thyroid hormone by certain phthalates observed in this study. Further studies are necessary to confirm and further elucidate the exact mechanism(s) behind our findings.

## Supporting Information

S1 Fig Chromatograms of phthalate metabolites in pregnant women’s urine, as measured by LC-MS/MS. (TIF)

S2 Fig Use of plastic or glass vial to collect urine will not affect the level of phthalate metabolites measured in the urine. (TIF)

S1 Table The native and labeled precursor and product ion transitions, retention time, and MRM-parameters of nine phthalate metabolites by a high performance liquid chromatography electrospray ionization tandem mass spectrometry. (DOCX)

S2 Table Quality control (spiked in urine) data and quantification limits of this method for the determination of nine phthalate metabolites in pregnant women’s urine. (DOCX)

S3 Table Median urinary concentration of phthalate metabolites (ng/mL) measured for pregnant women in different studies. (DOCX)

S4 Table Summary of human studies about phthalates exposure and thyroid function. (DOCX)
