# Supplementary figures and images for: Relationship of Urinary Phthalate Metabolites with Serum Thyroid Hormones in Pregnant Women and Their Newborns: A Prospective Birth Cohort in Taiwan
Source: PLoS One. 2015 Jun 4;10(6):e0123884. doi: 10.1371/journal.pone.0123884 (PMC4456348; doi:10.1371/journal.pone.0123884)

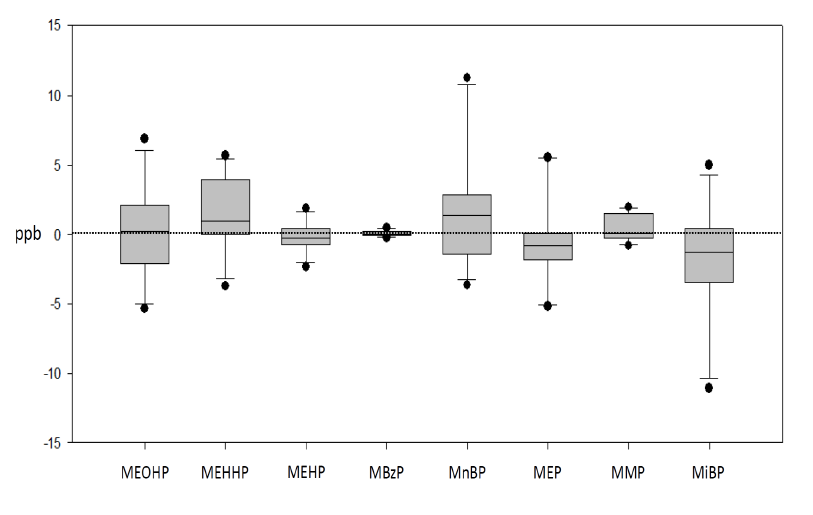

Supplement: S1 Fig — (TIF) [file pone.0123884.s001.tif]

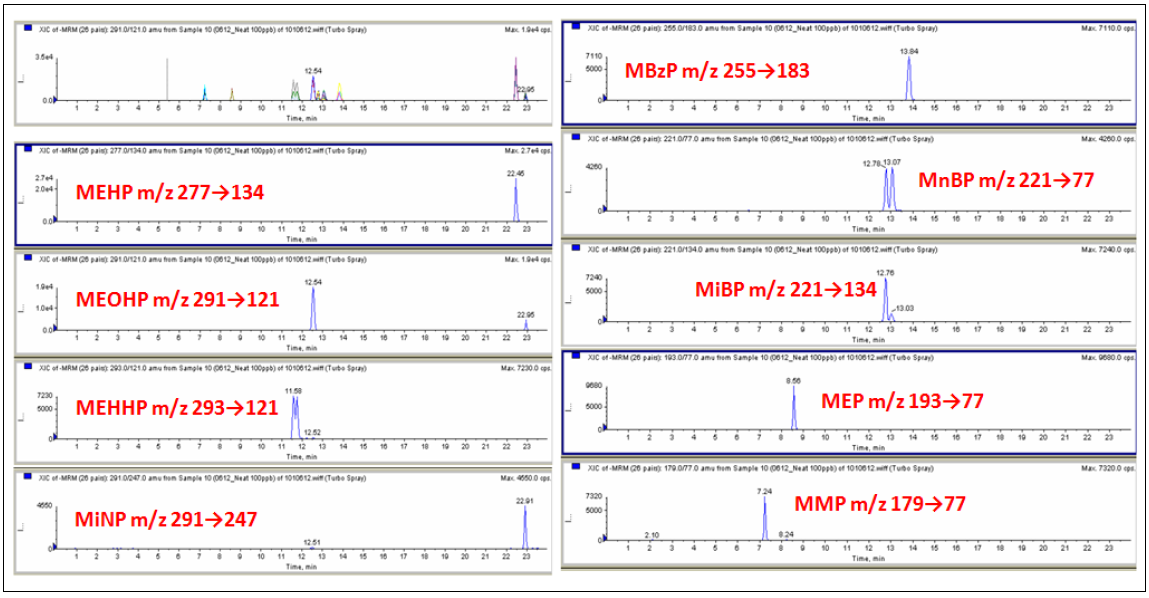

Supplement: S2 Fig — (TIF) [file pone.0123884.s002.tif]
