# Supplementary material for: Relationship of Urinary Phthalate Metabolites with Serum Thyroid Hormones in Pregnant Women and Their Newborns: A Prospective Birth Cohort in Taiwan
Source: PLoS One. 2015 Jun 4;10(6):e0123884. doi: 10.1371/journal.pone.0123884 (PMC4456348; doi:10.1371/journal.pone.0123884)
Supplement: S1 Table — (DOCX) [file pone.0123884.s003.docx]

**S1 Table.** **Native and labeled precursor and product ion transitions, retention time, and MRM-parameters of nine phthalate metabolites by a high-performance liquid chromatography electrospray ionization tandem mass spectrometry.**

| **Analyte** | **Native (Q1/Q3)** | **Labeled (Q1/Q3)** | **RT (min)** | **DP** | **EP** | **CE** | **CXP** |
| --- | --- | --- | --- | --- | --- | --- | --- |
| MEHP | 277/134 | 281/137 | 22.21 | -65 | -10 | -22 | -7 |
| MEHHP | 293/121 | 297/124 | 10.88 | -70 | -10 | -26 | -3 |
| MEOHP | 291/121 | 295/124 | 11.79 | -60 | -10 | -28 | -5 |
| MnBP | 221/77 | 225/79 | 12.32 | -60 | -10 | -28 | -1 |
| MiBP | 221/134 | - | 12.06 | -40 | -10 | -28 | -11 |
| MEP | 193/77 | 197/79 | 8.07 | -50 | -10 | -24 | -11 |
| MBzP | 255/183 | 259/107 | 13.03 | -60 | -10 | -18 | -9 |
| MMP | 179/77 | 183/79 | 6.85 | -45 | -10 | -26 | -1 |
| MiNP | 291/247 | 295/250 | 22.69 | -65 | -10 | -20 | -19 |

Abbreviation: MRM = Multiple reaction monitoring; RT = Retention time; DP = Declustering potential; EP = Entrance potential;

CE = Collision energy; CXP =Collision cell exit potential.
