# Supplementary material for: Relationship of Urinary Phthalate Metabolites with Serum Thyroid Hormones in Pregnant Women and Their Newborns: A Prospective Birth Cohort in Taiwan
Source: PLoS One. 2015 Jun 4;10(6):e0123884. doi: 10.1371/journal.pone.0123884 (PMC4456348; doi:10.1371/journal.pone.0123884)
Supplement: S2 Table — (DOCX) [file pone.0123884.s004.docx]

**S2 Table.** **Quality control (spiked in urine) data and quantification limits of this method for the determination of nine phthalate metabolites in pregnant women’s urine.**

| N = 5 | QC low | | | |  | QC high | | | |
| --- | --- | --- | --- | --- | --- | --- | --- | --- | --- |
|  | Spiked conc.  (ng/ml)  mean±SD | Accuracy  (%) | RSD (%)  Intra-day | RSD (%)  Inter-day |  | Spiked conc.  (ng/ml)  mean±SD | Accuracy  (%) | RSD (%)  Intra-day | RSD (%)  Inter-day |
| MEOHP | 9.91±0.24 | 99.1 | 2.1 | 8.4 |  | 95.24±2.03 | 95.2 | 2.3 | 6.3 |
| MEHHP | 10.31±0.36 | 103.1 | 8.6 | 6.1 |  | 99.36±1.22 | 99.3 | 1.4 | 4.5 |
| MEHP | 9.14±0.45 | 91.4 | 4.6 | 3.5 |  | 102.45±3.21 | 102.4 | 2.3 | 3.8 |
| MBzP | 9.68±0.33 | 98.6 | 3.0 | 7.4 |  | 101.23±2.51 | 101.2 | 3.4 | 8.6 |
| MnBP | 11.21±0.25 | 112 | 7.4 | 11.3 |  | 102.56±0.33 | 102.6 | 2.1 | 10.5 |
| MiBP | 9.34±1.32 | 93.4 | 2.8 | 3.2 |  | 101.43±3.12 | 101.4 | 2.6 | 2.5 |
| MEP | 9.87±0.81 | 98.7 | 0.5 | 6.5 |  | 95.65±2.87 | 95.7 | 8.6 | 7.9 |
| MMP | 10.46±0.12 | 104.6 | 0.7 | 2.7 |  | 97.16±0.65 | 97.2 | 4.7 | 5.4 |
| MiNP | 10.11±1.24 | 101.1 | 9.1 | 8.2 |  | 92.12±1.36 | 92.1 | 8.4 | 3.7 |

QC low and QC high: Pooled quality control urine spiked with 10 ng/ml and 100 ng/ml of each compound.
