# Supplementary material for: Relationship of Urinary Phthalate Metabolites with Serum Thyroid Hormones in Pregnant Women and Their Newborns: A Prospective Birth Cohort in Taiwan
Source: PLoS One. 2015 Jun 4;10(6):e0123884. doi: 10.1371/journal.pone.0123884 (PMC4456348; doi:10.1371/journal.pone.0123884)
Supplement: S3 Table — (DOCX) [file pone.0123884.s005.docx]

**S3 Table.** Median urinary concentration of phthalate metabolites (ng/mL) measured for pregnant women in different studies.

| **Country** | **Sampling year** | **Number of participants** | **DEHP** |  |  | **DEP** | **DnBP** | **DiBP** | **DiNP** | **BBzP** | **DMP** | **References** |
| --- | --- | --- | --- | --- | --- | --- | --- | --- | --- | --- | --- | --- |
|  |  |  | **MEHP** | **MEOHP** | **MEHHP** | **MEP** | **MnBP** | **MiBP** | **MiNP** | **MBzP** | **MMP** |  |
| Taiwan | 2009-2010 | 148 | 7.7 | 13.4 | 14.5 | 22.5 | 24.5 | 13.2 | <LOD | 0.99 | 5.4 | This study |
| Taiwan | 2001-2002 | 100 | 10.5 | 21.7 | 20.8 | - | 52.4 | 10.3 | <LOD | 1.2 | - | Wang et al. (2011) |
| Taiwan | 2005-2006 | 76 | 20.6 | - | - | 27.7 | 81.1 | - | - | 0.9 | 4.3 | Huang et al. (2007) |
| Japan | 2005-2006 | 50 | 4.0 | 10.6 | 11.0 | 7.8 | 57.9 | - | - | 3.7 | 6.6 | Suzuki et al. (2009) |
| USA | 2000 | 25 | 4.6 | - | - | 236.0 | 42.6 | - | - | 12.1 | - | Adibi et al. (2003) |
| USA | 1999-2002 | 241 | 3.3 | 11.4 | 11.1 | 128.4 | 13.5 | 2.5 | - | 8.3 | 0.7 | Swan et al. (2005) |
| USA | 1998-2002 | 404 | 6.0 | 20.0 | 17.0 | 380.0 | 36.0 | 6.2 | - | 22.0 | 1.6 | Wolff et al. (2008) |
| USA | 1998-2001 | 246 | 4.8 | 19.9 | 17.5 | 202.0 | 35.5 | 10.2 | - | 17.2 | - | Adibi et al. (2008) |
| USA | 2000-2004 | 283 | 3.5 | 11.2 | 9.9 | - | - | - | - | - | - | Adibi et al. (2009) |
| USA | 2003-2004 | 150 | 114.7 | 108.9 | 95.1 | 168.5 | 14.8 | 4.6 | - | 9.2 | - | Yan et al. (2009) |
| France | 2007 | 279 | 16.7 | 41.9 | 28.5 | 43.6 | 35.7 | 53.7 | < 4 | 10.1 | - | Zeman et al. (2013) |
| Netherlands | 2004-2006 | 99 | 6.9 | 14.0 | 14.5 | 117.0 | 42.7 | 42.1 | - | 7.5 | - | Ye et al. (2008) |
| Israel | 2006 | 19 | 6.8 | 21.5 | 17.5 | 165.0 | 30.8 | 15.6 | - | 5.3 | - | Berman et al. (2009) |
| Germany | 2008 | 11 | - | 10.5 | 12.0 | - | 23.7 | 33.6 | - | 5.0 | - | Wittassek et al. (2009) |
