# Supplementary material for: Relationship of Urinary Phthalate Metabolites with Serum Thyroid Hormones in Pregnant Women and Their Newborns: A Prospective Birth Cohort in Taiwan
Source: PLoS One. 2015 Jun 4;10(6):e0123884. doi: 10.1371/journal.pone.0123884 (PMC4456348; doi:10.1371/journal.pone.0123884)
Supplement: S4 Table — (DOCX) [file pone.0123884.s006.docx]

**S4 Table. Summary of human studies concerning phthalates exposure and thyroid function.**

| **First author/ Publication year** | **Type of study** | **Study period/ Country** | **Study subjects** | **Exposure** | **Outcome** | **Main findings** |
| --- | --- | --- | --- | --- | --- | --- |
| **Pregnant women** |  |  |  |  |  |  |
| Huang, 2007 | Cross-sectional study | 2005-2006/ Taiwan | 76 pregnant women required to undergo amniocentesis (> 35 yrs or abnormal serum AFP or β-HCG | 5 phthalate metabolites (MnBP, MBzP, MEHP, MEP, MMP) in one-spot urine of second trimester | TSH, T3, T4, FT4 in maternal serum of second trimester | Urinary MBP levels was negatively associated with FT4 and T4 (β = -0.110, P < 0.001 and β = -0.112, P = 0.003 respectively). |
| **This study** | **Cross-sectional study** | **08/2009-12/2010/Taiwan** | **148 healthy pregnant women (16-42 yrs)** | **9 phthalate metabolites (MEHP, MnBP, MiBP, MBzP, MEP, MMP, MiNP, MEHHP, MEOHP) in one-spot urine of third trimester** | **1.TSH, T3, T4, FT4 in maternal serum of third trimester**  **2. TSH, T3, T4, FT4 in serum cord blood** | **Urinary MBzP levels was significantly and negatively associated with TSH levels in cord blood serum (β= -2.604, P = 0.002).** |
| **Children** |  |  |  |  |  |  |
| Boas, 2010 | Cohort study | 2006/1-2007/8/ Denmark | 845 children (503 males and 342 females; 4-9 yrs) | 12 phthalate metabolites (MEP, MnBP, MBzP, MEHP, MEHHP, MEOHP, MECPP, MOP, MiNP, MHiNP, MOiNP, MCiOP) in one-spot urine | TSH, T3, T4, FT3, and FT4 in serum | Urinary MEP levels were significantly and negatively associated with T3 in girls (β= -0.11, P = 0.026) |
| Wu, 2013 | Cross-sectional & follow-up studies | 05/2011-06/2011/Taiwan | 60 children  (45 males and 15 females; ≤10 yrs) by intake of DEHP-tainted foodstuffs | Questionnaire for the intake of DEHP (median: 0.05 mg/kg/day; range: 0.002-0.19 mg/kg/day) | 1.TSH, T3, T4, FT3, and FT4 in baseline serum  2. TSH, T3, T4, FT3, and FT4 in follow-up serum 6 months later | 1. Serum TSH levels were negatively associated with daily intake of known DEHP-tainted foods (Spearman correlation coefficient r = 0.422, P = 0.0048  2. Serum T3 levels was significantly changed (P = 0.034) in high exposure group (n = 13). |
| **Adults or adolescents** |  |  |  |  |  |  |
| Rais-Bahrami, 2004 | Follow-up study | -^1^/ USA | 19 adolescents (13 males and 6 females; 14-16 yrs) | High DEHP exposures (4.2-14.0 mg kg/day) as neonates from intravenous form by extracorporeal membrane oxygenation (ECMO) | TSH, T4, and FT4 in serum of adolescents | Thyroid functions were within normal range for age and sex distribution. |
| Meeker, 2007 | Cross-sectional study | 01/2000-05/2004/USA | 408 men who visited infertility clinic (18–55 yrs) | 6 phthalate metabolites (MEP, MBP, MBzP, MEHP, MEHHP, MEOHP) in one-spot urine | TSH, FT4, and T3 in serum | An inverse association between urinary MEHP and serum FT4 and T3. |
| Meeker, 2011 | Cross-sectional study | 2007-2008/USA | 1,346 adults  (≥ 20 years) and 329 adolescents (12-19 years) from 2007-2008 NHANES | 7 phthalate metabolites (MEHP, MEHHP, MEOHP, MECPP, MnBP, MiBP, MCPP) in one-spot urine | TSH, T4, FT4, T3, and thyroglobulin in serum | 1.In adults, MEHHP displayed monotonic dose-dependent decreases in T4  2. In adolescents, a significant and positive association between DEHP secondary metabolites and T3 and TSH was demonstrated. |

Abbreviation described in Table 2; AFP = α-fetoprotein; β-HCG = β-human chorionic gonadotropin; NHANES = National Health and Nutrition Examination Survey.

^1^No information.
